# Clinicopathologic features and therapy outcome in childhood Hodgkin’s lymphoma: a report from tertiary care center in Saudi Arabia

**DOI:** 10.1186/s43046-021-00078-0

**Published:** 2021-08-16

**Authors:** Nawaf Alkhayat, Mohammad Alshahrani, Ghaleb Elyamany, Qanita Sedick, Walid Ibrahim, Hasna Hamzi, Amal Binhassan, Mohamed Othman, Saeed Alshieban, Mansour S. Aljabry, Shuaa Asiri, Muneerah Alzouman, Omar Alsuhaibani, Fahad Alabbas, Omar Alsharif, Yasser Elborai

**Affiliations:** 1 Department of Pediatric Hematology/Oncology, Prince Sultan Military Medical City, Riyadh, Riyadh, 11159 Saudi Arabia; 2 Department of Central Military Laboratory and Blood Bank, Prince Sultan Military Medical City, Riyadh, Saudi Arabia; 3 Department of Pathology and Laboratory Medicine, Ministry of National Guard Health Affairs, King Abdullah International Medical Research Center, King Saud bin Abdulaziz University for Health Sciences, Riyadh, Saudi Arabia; 4 Pathology Department, King Khalid University Hospital, King Saud University, Riyadh, Kingdom of Saudi Arabia; 5 Department of Pediatric Oncology, National Cancer Institute, Cairo University, Cairo, Egypt

**Keywords:** Pediatric Hodgkin lymphoma, Long-term outcome, Late toxicity, Saudi Arabia

## Abstract

**Background:**

Hodgkin lymphoma (HL) is lymphoid neoplasm usually affecting lymphatic system; it accounts 3.6% of cancers in Saudi Arabia. Modern treatment protocols had shown particular success rates in overall-survival (OS) and event-free-survival (EFS). In our study, we reviewed the medical records of 80 pediatric and young adolescent patients diagnosed HL from January 2006 to July 2020, treated at tertiary care hospital in Riyadh, Saudi Arabia. Demographic, clinical, and pathological data were explored. First line therapy was ABVD, COG, COPP, R-CHOP, or radiotherapy alone in 53/80 (66.4%), 24/80 (30%), 1/80 (1.2%), 1/80 (1.2%), or 1/80 (1.2%) patients; respectively. Response assessment was done by CT + / − PET scan after first 2 cycles then every 2 cycle and end of therapy. Another assessment was done if any clinical suspicion of recurrence.

**Results:**

Median age 11 (range 3–16) years. Males to females 1.3:1. Seventy-two out of eighty (90%) patients showed first complete remission (CR1) and maintained remission for median 40 (range 7–136) months. Eight out of eighty (10%) patients showed refractory disease. Nineteen patients received salvage therapy (ICE or ESHAP/brentuximab vedotin or gemcitabine/brentuximab vedotin), 14/19 (73.7%) had 2nd complete remission (CR2) for median time 24 (ranged 9–78) months, while 5/19 (26.3%) did not show any response. Five-year OS and EFS were 95% and 75%. Two patients had 2ry malignant neoplasms, one had AML and died, the other had malignant fibrous histocytoma and still alive. None of our patients had fertility problem. Also, they did not experience chronic pulmonary or cardiotoxicity. Classic Hodgkin’s lymphoma: nodular sclerosis subtype was more prominent (55%) than mixed cellularity subtype (22.5%), which is similar to several European and US studies, lymphocyte rich (11.25%) and lymphocyte depleted (0%), while nodular lymphocyte predominant Hodgkin’s lymphoma (11.25%).

**Conclusions:**

Our study provided unique descriptive study of childhood HL, in Saudi Arabia, with valuable insight into the long-term outcome and late toxicity. Our results are comparable to other studies in the Middle East and European countries.

## Background

Hodgkin lymphoma (HL) is lymphoid neoplasm usually affecting lymph nodes and the lymphatic system; it is considered to be among the hematological malignancies with the best prognosis [1]. In childhood and young adolescence, HL has shown to display heterogeneous clinical and pathological manifestations and appears to vary according to the epidemiological and geographic origin of the disease [2]. Some studies have also found HL manifestations to be correlated to socio economic levels and immunologic status [3, 4].

HL accounts for 30% of all lymphomas and 5–6% of all childhood cancers [5]. In the USA, it accounts for approximately 7% of childhood cancers and 1% of childhood deaths. And in developed countries, HL accounts for approximately 10% of all lymphomas and 0.6% of all cancer diagnoses [6], while HL accounts for 3.6% of all cancers in Saudi Arabia [7].

Data shows that the number of Hodgkin lymphoma (HL) in the Middle East has increased over the past decade especially in younger age groups [8], in parallel to reduced HL associated mortalities worldwide between 2005 and 2015 [9]. Improvement in living conditions, healthcare systems, and modern treatment protocols have contributed to improved mortality [10]. Indeed, the modern treatment protocols for HL has shown particular success rates in children with the 5-year event-free survival (EFS) in childhood and adolescence exceeding 90% for early onset disease and 80% for late onset advanced stage disease [11].

To the best of our knowledge, there is a paucity of data available which describes the clinic-pathological features and survival rate of childhood and adolescent HL in the Middle East and Saudi Arabia. The recent increased prevalence of HL occurring in children and young adults [8] prompted us to review the clinic-pathological data and survival outcome of HL in Saudi Arabia.

## Methods

### Study population

The data were reviewed, retrospectively, through the medical records of 89 pediatric and young adolescent patients diagnosed as HL between the period January 2006 and July 2020 treated in our tertiary institution in Riyadh, Saudi Arabia. The study was approved by the research and ethics committee with the institute review board (IRB) number 1390 (HP-01-R079).

The patients’ records were searched for all demographic, clinical, radiological, laboratory, treatment protocol, and follow up data. The diagnosis of HL was confirmed histologically by examination of a lymph node biopsy/tissue and confirmed with immunohistochemical stains according to the World Health Organization (WHO) classification [12]. All patients were assessed physically and radiologically. They all had contrast-enhanced computed tomography (CT) scan of the neck, chest, abdomen, and pelvis initially for staging. Positron emission tomography (PET) scan was performed initially for most of the patients; few patients missed it because of technical issues. Response assessment by CT + / − PET scan was performed after first 2 cycles then every 2 cycles and end of therapy. If PET scan was not available, we built up the response criteria upon the results of CT according to the protocol. Bone marrow aspiration and trephine biopsies were performed on the selected patients to diagnose bone marrow metastasis if clinically indicated, stage III/IV or low parameters of complete blood count (CBC) which may indicate bone marrow involvement.

Our patients were staged according to the Cotswold modification of the Ann Arbor staging [13]. Risk assessment was performed at diagnosis: stages IA and IIA without bulky disease or extra nodal extension were considered as low-risk; stage IA or IIA with bulky disease or extra nodal extension, stage IB or IIB, and stage IIIA were considered as intermediate risk, while stages IIIB and IV were considered as high risk [14]. Bulky disease was defined as a single node > 6 cm or a multiple amalgamated nodes tumor mass of > 10 cm. Early stage considered as stages I and II, while advanced stage considered as stages III and IV. Response assessment and follow-up were based on the Cheson criteria [15].

### Treatment protocols and follow-up policy

Different protocols were used as 1st line of treatment. Fifty-three patients received doxorubicin (adriamycin), bleomycin, vinblastine, and dacarbazine (ABVD) in the period between the beginning of the study in 2006 till end of 2015, 2 cycles of ABVD for low risk, 4 cycles for intermediate risk, and 6 cycles for high risk. Twenty-four patients received Children’s Oncology Group (COG) protocols (low risk AHOD0431, intermediate risk AHOD0031, and high risk AHOD0831) in the period between 2016 till end of study in July 2020. One patient, high risk stage IIIB, received cyclophosphamide, vincristine, procarbazine, prednisone (COPP) due to severe heart failure. Another patient, intermediate risk stage IIIA, received rituximab-cyclophosphamide, hydroxydaunomycin, vincristine, and prednisolone (R-CHOP), Although this chemotherapy was not the standard of care, but the decision was taken by primary physician based on pathology subtype, nodular lymphocyte predominant Hodgkin’s lymphoma. Only one patient received radiotherapy alone as 1st line therapy, because his risk stratification was low risk stage IA, the primary physician took this decision although it is not the standard of care. Unfortunately, he relapsed and went in remission after salvage therapy.

Involved filed radiotherapy (IFRT) was given to 41/80 (53.3%) patients after completion of different chemotherapy protocols (ABVD & COG) according to the risk assessment, bulky disease, and response to treatment. For low risk patients, IFRT was given to all patients who did not have a complete response (CR) after receiving 3 cycles of chemotherapy. For intermediate risk patients, IFRT was given to; slow early responders (SER) who had less than very good partial response (< VGPR, < 60% reduction of initial volume) following 2 cycles of chemotherapy. Also, IFRT was given to rapid early responders (RER) who had CR or VGPR following 2 cycles of chemotherapy but failed to get CR after 4 cycles of chemotherapy, while IFRT was omitted to RER following 2 cycles of chemotherapy and successfully got CR after 4 cycles of chemotherapy. For high risk patient, IFRT was risk-adapted with an intended volume reduction to reduce toxicity. RER who had CR after first 2 cycles of chemotherapy, only got IFRT to the initial bulk disease. While SER who had partial response (50% of initial volume) or stable disease (> 50% of initial volume), sites that remain PET avid after the initial 2 cycles chemotherapy will be irradiated as well as any site (regardless of avidity) that remains ≥ 2.5 cm at completion of all chemotherapy. The dose of IFRT was 21 Gy in 14 fractions of 1.5 Gy per day. Treatment began within 3–4 weeks after completion of the last cycle of chemotherapy provided that recovered blood counts. Response criteria depended on results of PET scan. In 9/80 patients who did not have PET scan due to technical issues, minimal two perpendicular dimensions in CT scan were used to determine the response criteria.

Second line of chemotherapy was given to the patients who had refractory or relapsed disease. Refractory disease was defined as stable disease after completion of treatment protocol (50% of the initial volume of LNDs) with viable cell confirmed by histopathology, or progressive disease at any time during treating protocol, confirmed by histopathology. While relapsed disease was defined as recurrence of disease after variable periods of complete remission post first line therapy, the second line therapy includes different protocols of chemotherapy: ICE (ifosfamide, carboplatin, etoposide) or ESHAP (etoposide, solu-medrol, high dose ara-c, platinum “cisplatin”)/brentuximab vedotin, or gemcitabine/brentuximab vedotin. If the patient got in remission by second line chemotherapy, this remission was consolidated by high dose chemotherapy and autologous stem cell rescue.

Patients underwent follow-up with clinical examinations, chest X-ray, and abdominal ultrasound at 3-month intervals for the first 3 years, then at 6-month intervals till 5 years, and then yearly. More investigations on follow-up were performed only if indicated.

### Statistical analysis

The data was statistically described using number (percent). Association between variables and groups is performed using chi-square test or Fisher exact test. Overall and event-free survival curves are calculated using Kaplan–Meier test. Statistical package for social sciences (SPSS) computer program (version 19 windows) was used for data analysis. *P* value ≤ 0.05 was considered significant.

## Results

We collected data from 89 pediatric patients diagnosed as HL, 9 cases were excluded due to incomplete data or lost follow up, 80 patients were admitted in our tertiary care institution in Riyadh Saudi Arabia with confirmed HL during the period between January 2006 and July 2020. The median age was 11 years old with range 3–16 years. The cohort study included 45 males and 35 females with 1.3:1 male to female ratio. Laboratory data and other clinical data; B symptoms (fever, weight loss, and night sweats), primary site, bulky disease, extra nodal involvement, presence of Epstein-Barr virus, pathology subtypes, stages, and risk groups, were presented in Table 1.

**Table 1:** Clinical and demographic data

	No	%
Age (years)		
< 5	3	3.7
5–9.99	23	28.8
≥ 10–16	54	67.5
Gender		
Male	45	56.3
Female	35	43.7
B symptoms		
No	46	57.5
Yes	34	42.5
Primary site		
Peripheral lymph nodes	65	81.25
Mediastinum	15	18.75
Bulky		
No	59	73.8
Yes	21	26.2
Extra nodal involvement		
No	51	63.8
Yes	29	36.2
Epstein-Barr virus		
Positive	40	50
Negative	5	6.3
N/A	35	43.7
Pathology		
Classic Hodgkin’s lymphoma		
Lymphocyte rich	9	11.25
Mixed cellularity	18	22.5
Nodular sclerosis	44	55
Lymphocyte depleted	0	0
Nodular lymphocyte predominant Hodgkin’s lymphoma	9	11.25
Stage		
I	5	6.3
II	25	31.2
III	42	52.5
IV	8	10
Early and advanced stages		
Early (I, II)	30	37.5
Advanced (III, IV)	50	62.5
Risk group		
Low	12	15
Intermediate	44	55
High	24	30
Laboratory data: median (range)		
WBC (× 10^9^/l)	7.4 (2.3–31.6)	
HGB (× 10^9^/l)	11.8 (6.1–15.2)	
PLT (× 10^9^/l)	357 (27–856)	
LDH (μ/l)	266.5 (131–1164)	

*WBC* white blood cells, *HGB* hemoglobin, *PLT* platelet, *LDH* lactate dehydrogenase

### Response to therapy

All patients received 1st line of treatment which was chemotherapy alone, radiotherapy alone, or combination of both chemotherapy/radiotherapy; 38/80 (47.5%), 1/80 (1.2%), or 41/80 (51.3%), respectively. The first line chemotherapy was ABVD, COG, COPP, or R-CHOP in 53/80 (66.4%), 24/80 (30%), 1/80 (1.2%), or 1/80 (1.2%) patients, respectively. Most of the patients 72/80 (90%) showed complete remission after first line therapy (CR1) and maintained remission for median 40 months and range 7–136 months, while 8/80 (10%) patients did not show any response, refractory disease.

Nineteen patients received second line (salvage) therapy, eight patients were refractory and eleven patients relapsed after median period of maintaining remission 30 months ranged 3–96 months. The salvage therapy was either chemotherapy alone 7/19 (36.8%) or chemotherapy followed by stem cell rescue 12/19 (63.2%). Response to salvage therapy showed 14/19 (73.7%) patients had 2nd complete remission (CR2) for median time 24 months ranged (9–78) months, while 5/19 (26.3%) patients did not show any response (Table 2).

**Table 2:** Salvage treatment and outcome of all refractory/relapsed patients

# of patient	Second line of chemotherapy	HSCT	Response	Outcome
Refractory patients				
1	ICE	Yes	RD	Died from disease
2	ESHAP/brentuximab vedotin	Yes	CR2	Alive in CR
3	ICE	Yes	CR2	Alive in CR
4	ESHAP/brentuximab vedotin	Yes	CR2	Alive in CR
5	ICE	No	RD	Died from disease
6	ICE then ESHAP/brentuximab vedotin	No	RD	Alive with disease
7	ICE	Yes	CR2	Alive in CR
8	ICE then ESHAP/brentuximab vedotin	Yes	CR2	Alive in CR
Relapsed patients				
1	ICE then gemcitabine/brentuximab vedotin	No	RD	Died from disease
2	ICE	No	CR2	Alive in CR
3	ICE	No	CR2	Alive in CR
4	ICE	No	CR2	Alive in CR
5	ICE	Yes	CR2	Alive in CR
6	ICE	Yes	CR2	Alive in CR
7	ESHAP/brentuximab vedotin	Yes	CR2	Alive in CR
8	ICE	Yes	CR2	Alive in CR
9	Refused treatment	No	RD	Alive with disease
10	ICE then gemcitabine/brentuximab vedotin	Yes	CR2	Alive in CR
11	ESHAP/brentuximab vedotin	Yes	CR2	Alive with 2ry neoplasm

*ICE* ifosfamide/carboplatin/etoposide, *ESHAP* etoposide/solu-medrol/high dose ara-c/platinum-cisplatin, *HSCT* hematopoietic stem cell transplant, *CR2* second complete remission, *CR* complete remission, *RD* refractory disease

Table 3 presents a number of clinical factors and their correlation with CR1. Statistical analysis of these factors revealed that B symptoms, stage, and risk groups have significant correlation with remission rate after 1st line therapy, *P* = 0.05, *P* = 0.022, *P* = 0.012, respectively. While other factors, age, gender, primary site, bulky disease, extra nodal involvement, and pathology subtypes, did not show any clinical significant with rate of CR1.

**Table 3:** Factors affecting the response to 1st line therapy

	CR1 (*n* = 72)	Non-CR1 (*n* = 8)	*P* value
Age (years)			
< 5 years (*n* = 3)	3 (4.2%)	0 (0.0%)	0.118
5–9.99 years (*n* = 23)	23 (31.9%)	0 (0.0%)	
≥ 10 years (*n* = 54)	46 (63.9%)	8 (100.0%)	
Gender			
Female (*n* = 35)	32 (44.4%)	3 (37.5%)	0.707
Male (*n* = 45)	40 (55.6%)	5 (62.5%)	
B symptoms			
No (*n* = 46)	44 (61.1%)	2 (25.0%)	0.050*
Yes (*n* = 34)	28 (38.9%)	6 (75.0%)	
Primary site			
Peripheral lymph nodes (*n* = 65)	58 (80.6%)	7 (87.5%)	0.286
Mediastinum (*n* = 15)	14 (19.4%)	1 (12.5%)	
Bulky			
No (*n* = 59)	52 (72.2%)	7 (87.5%)	0.351
Yes (*n* = 21)	20 (27.8%)	1 (12.5%)	
Extra nodal involvement			
No (*n* = 51)	48 (66.7%)	3 (37.5%)	0.104
Yes (*n* = 29)	24 (33.3%)	5 (62.5%)	
Pathology			
cHL, nodular sclerosis (*n* = 44)	39 (54.2%)	5 (62.5%)	0.395
cHL, mixed cellularity (*n* = 18)	15 (20.8%)	3 (37.5%)	
cHL, lymphocyte rich (*n* = 9)	9 (12.5%)	0 (0.0%)	
NLPHL (*n* = 9)	9 (12.5%)	0 (0.0%)	
Stage			
Early (I, II) (*n* = 30)	30 (41.7%)	0 (0.0%)	0.022*
Advanced (III, IV) (*n* = 50)	42 (58.3%)	8 (100.0%)	
Risk group			
Low (*n* = 12)	12 (16.7%)	0 (0.0%)	0.012*
Intermediate (*n* = 44)	42 (58.3%)	2 (25.0%)	
High (*n* = 24)	18 (25.0%)	6 (75.0%)	

Data are expressed as number (%); *p* > 0.05 = not significant; *p* ≤ 0.05 = significant. *CR1* complete remission after 1st line of therapy, *Non-CR1* not in complete remission after 1st line of therapy, *cHL* classic Hodgkin’s lymphoma, *NLPHL* nodular lymphocyte predominant Hodgkin lymphoma

Table 4 presents group of patients 19/80, who are refractory to 1st line therapy or relapsed after CR1. Univariate analysis of initial presentation shows factors that have significant correlation with this relapsed/refractory (R/R) group. B symptoms and risk groups have a significant correlation with relapsed/refractory group, *P* = 0.037 and 0.009, respectively. Other clinical factors did not show any significant correlation with relapsed/refractory (R/R) group.

**Table 4:** Factors affecting the relapse/refractory (R/R) rate

	Not R/R (*n* = 61)	R/R (*n* = 19)	*P* value
Age (years)			
< 5 years (*n* = 3)	3 (4.2%)	0 (0.0%)	0.061
5–9.99 years (*n* = 23)	21 (34.4%)	2 (10.5%)	
≥ 10–16 years (*n* = 54)	37 (60.7%)	17 (89.5%)	
Gender			
Female (*n* = 35)	26 (42.6%)	9 (47.4%)	0.716
Male (*n* = 45)	35 (57.4%)	10 (52.6%)	
B symptoms			
No (*n* = 46)	39 (63.9%)	7 (36.8%)	0.037*
Yes (*n* = 34)	22 (36.1%)	12 (63.2%)	
Primary site			
Peripheral lymph nodes (*n* = 65)	51 (83.6%)	14 (73.7%)	0.422
Mediastinum (*n* = 15)	10 (16.4%)	5 (26.3%)	
Bulky			
No (*n* = 59)	45 (73.8%)	14 (73.7%)	0.994
Yes (*n* = 21)	16 (26.2%)	5 (26.3%)	
Extra nodal involvement			
No (*n* = 51)	40 (65.6%)	11 (57.9%)	0.543
Yes (*n* = 29)	21 (34.4%)	8 (42.1%)	
Pathology			
cHL, nodular sclerosis (*n* = 44)	35 (57.4%)	9 (47.4%)	0.827
cHL, mixed cellularity (*n* = 18)	13 (21.3%)	5 (26.3%)	
cHL, lymphocyte rich (*n* = 9)	6 (9.8%)	3 (15.8%)	
NLPHL (*n* = 9)	7 (11.5%)	2 (10.5%)	
Stage			
Early (I, II) (*n* = 30)	26 (42.6%)	4 (21.1%)	0.090
Advanced (III, IV) (*n* = 50)	35 (57.4%)	15 (78.9%)	
Risk group			
Low (*n* = 12)	11 (18.0%)	1 (5.3%)	0.009*
Intermediate (*n* = 44)	37 (60.7%)	7 (36.8%)	
High (*n* = 24)	13 (21.3%)	11 (57.9%)	

Data are expressed as number (%); *p* > 0.05 = not significant; *p* ≤ 0.05 = significant

*cHL* classic Hodgkin’s lymphoma, *NLPHL* nodular lymphocyte predominant Hodgkin’s lymphoma

### Long-term outcome and late toxicity

A total of 76/80 (95%) patients were alive. Seventy patients were alive in complete remission without any late toxicity. Two patients were alive with disease. One patient developed chemotherapy related toxicity which is moderate loss of hearing acuity after 4 cycles of ICE salvage therapy. Three patients developed complications within the field of radiotherapy; one patient had secondary neoplasm of malignant fibrous histocytoma at neck of left femur which was the metastatic site of initial disease and received local radiotherapy on it; two patients received IFRT on neck, one developed nasopharyngeal mass showed reactive lymphoid hyperplasia, and the other one developed hypothyroidism. None of our patients complained of any fertility problem. Also, they did not experience chronic pulmonary or cardiac toxicity.

Four patients died 4/80 (5%), 3 patients died of disease progression and one died of 2ry neoplasm. The three patients who died of disease progression; 2 of them were high risk HL (stage III B non-bulky) refractory to the first line therapy and did not respond to the second line therapy and died of disease, the third patient also was high risk HL (stage III B non-bulky), he responded to the first line therapy ABVD and maintained remission for 96 months but he relapsed and did not respond to 2nd line therapy and died of disease. The patient who died of 2ry neoplasm, he was diagnosed as intermediate risk HL (stage IIIA, non-bulky), he received ABVD with IFRT, he was in remission for 54 months but developed 2ry acute myeloid leukemia (AML) and died of 2ry neoplasm.

**Fig. 1:**
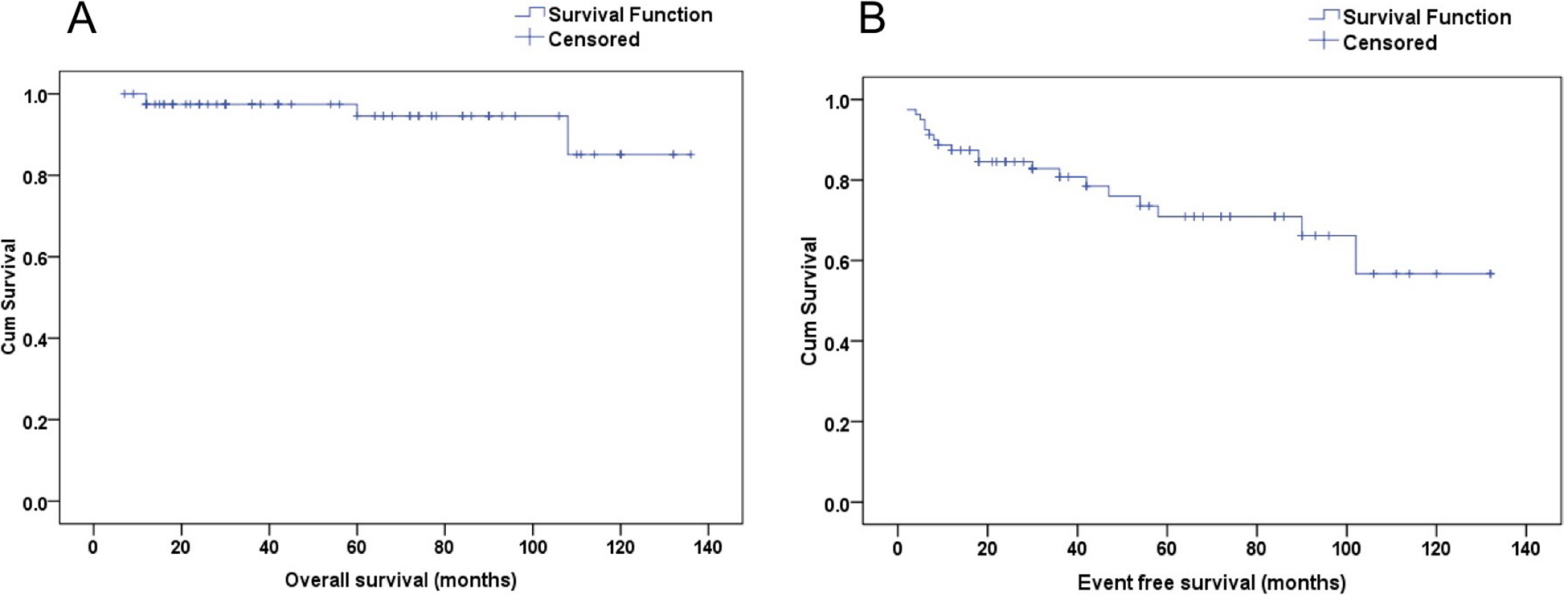
(**A**) Five-year overall survival. (**B**) Five-year event-free survival of Hodgkin’s lymphoma pediatric patients

**Fig. 2:**
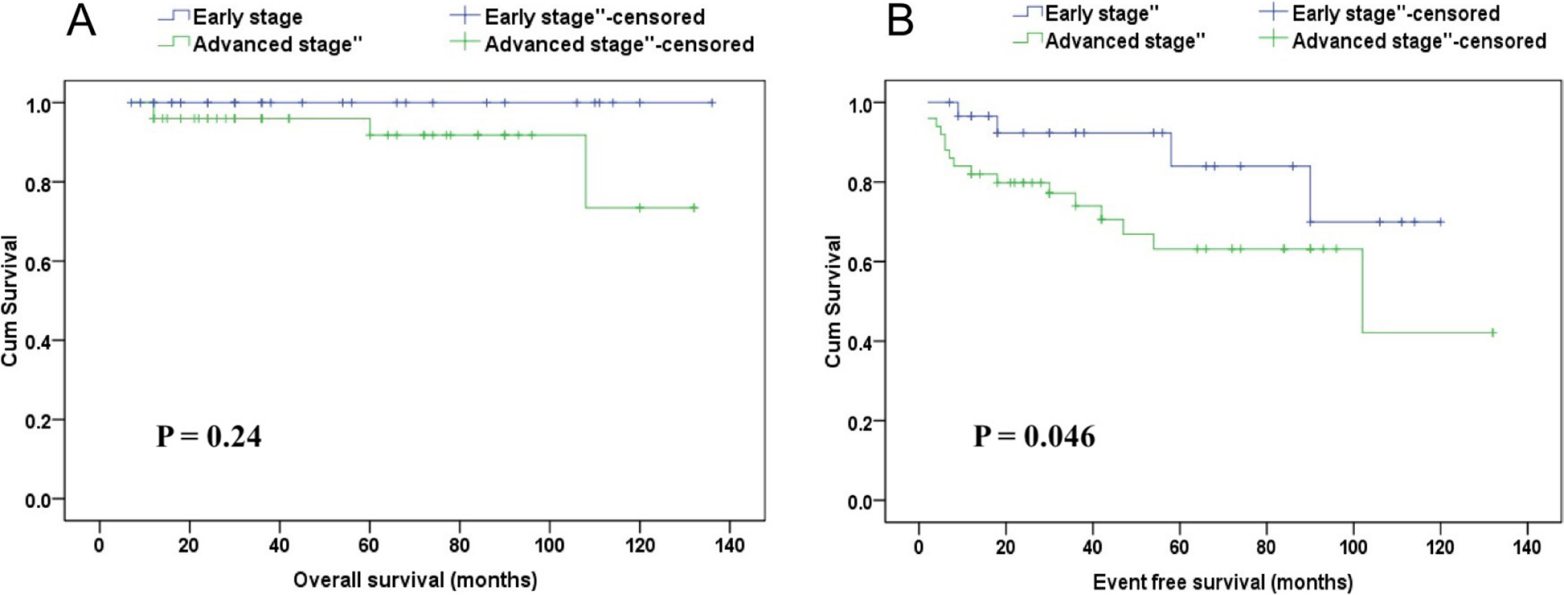
(**A**) Stage-related overall survival. (**B**) Stage-related event-free survival of Hodgkin’s lymphoma pediatric patients

**Fig. 3:**
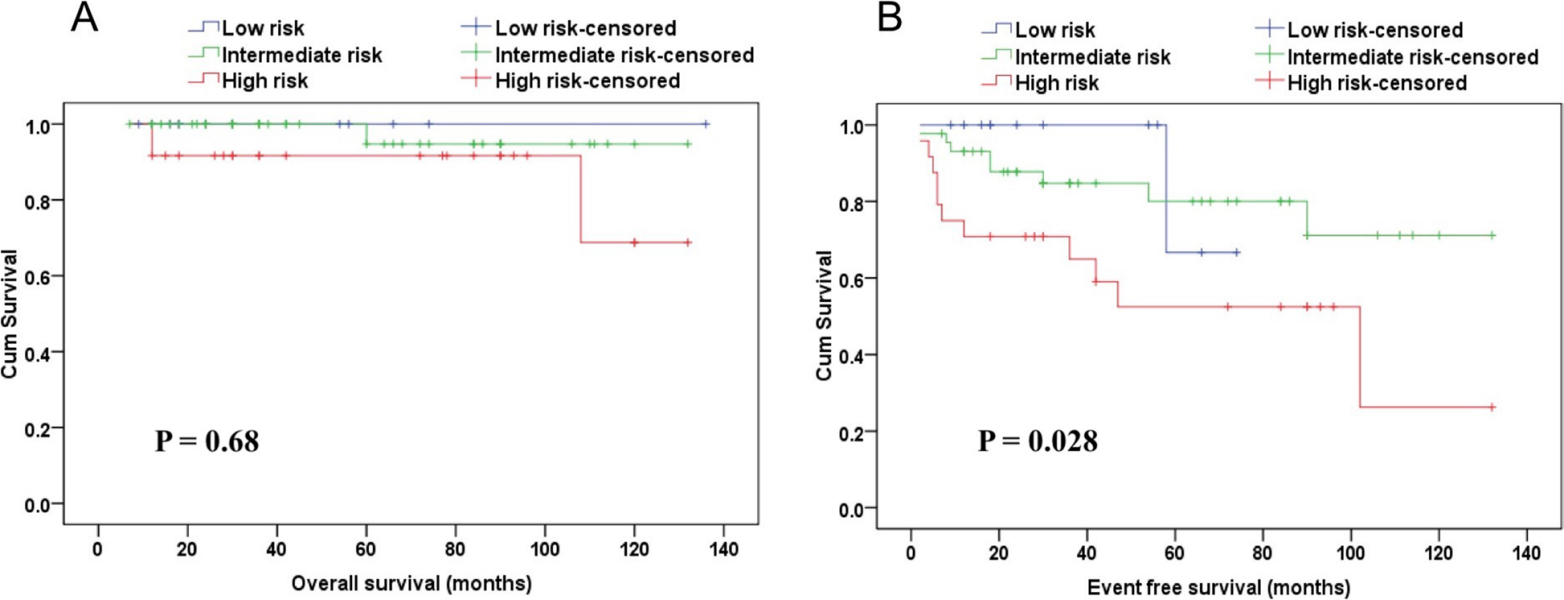
(**A**) Risk-related overall survival. (**B**) Risk-related event-free survival of Hodgkin’s lymphoma pediatric patients

The overall 5-year survival (OS) rates and event-free survival (EFS) rate of our study were 95% and 75%, respectively (Fig. 1 A and B). Univariate analysis of early (I and II) and advanced (III and IV) stages showed that, OS was 100% and 92% without statistical significance (*p* = 0.24), while EFS was 87% and 68%, respectively, with borderline significance *p* = 0.046 (Fig. 2 A and B). Also, univariate analysis of risk factor (low, intermediate, and high) showed OS 100%, 97%, and 87%, respectively (*p* = 0.68), while EFS 91%, 81%, and 54%, respectively, with significant *p* = 0.028 (Fig. 3 A and B).

## Discussion

HL is considered a highly curable disease among other malignancies; it has a worldwide clinical and epidemiological variation [16]. Our study discussed the epidemiological, clinical characteristics, and current management strategies with special attention to long-term outcome and late toxicity of pediatric HL in one of the biggest referral center in Saudi Arabia.

Our cohort revealed male: female ratio 1.3:1; this male predominance and other clinical feature like older age at presentation were reported in many other studies [17, 18]. Nodal presentation, in our cohort, is more prominent than extra-nodal presentation, 63.8% and 36.2% respectively, which are the same clinical features reported by Sherief et al. [17].

In our cohort, advanced or extensive disease (stages III-IV) showed a higher percentage than early or localized one (stages I-II); they were 62.5% and 37.5% respectively. Other countries with low socioeconomic level showed a similar percentage [19], Sherief et al. showed a higher percentage of advanced stage 56% in comparison to the early stage 44% [17]. In contrast, the European countries with higher socioeconomic level showed a lower percentage of advanced disease, may be due to early diagnosis, Aquino et al. showed early and advanced stages, 70% and 30%, respectively [16].

According to WHO, HL classified into classic HL (cHL) represented 95% of all HL which incorporate 4 subtypes; nodular sclerosis (NS), mixed cellularity (MC), lymphocyte rich (LR) and lymphocyte depleted (LD), and nodular lymphocyte predominant Hodgkin’s lymphoma (NLPHL) represented 5% of all HL [20]. In our cohort, the percentage of cHL and NLPHL were 88.7% and 11.3%; respectively. NS subtype was more prominent than MC, 55% and 22.5% respectively, which is similar to several European and US studies [16, 21]. On the other hand, some developing countries showed that MC subtype was more prominent than NS. In Egypt, MC and NS were 50.8% and 28.9% respectively [17]; in Nicaragua MC and NS, subtypes were 52.1% and 31% respectively [19], and in India MC subtype was 71% [22]. No cases detected with LD among our cohort.

The pathogenesis of HL in childhood has been largely attributed to viral oncogenic pathway stimulation. Epstein-Barr virus (EBV) has been identified, serologically by detecting IgG and IgM, as the most common associated virus. Genetic predisposition has also been associated in some cases [23]. The majority of patients in the cohort (50%) were positive for Epstein-Barr virus (EBV).

HL characterized by a higher curative rate and lower late toxicity among other malignancies especially in childhood. Our study showed high 5-year OS and EFS, 95% and 75%, respectively, which is similar to Turkish study reported by Uysal et al., the OS and EFS were 96% and 72%, respectively [24]. An Egyptian study reported by Sherief et al. showed OS and EFS, 96.6% and 84.7%, respectively [17], and Greek study reported by Pourtsidis et al. showed OS and EFS, 98% and 86.2%, respectively [21].

Survival rate was significantly affected by few clinical and pathological factors, B symptoms, stage, and risk group. In our study, 5-year survival rate was significantly lower in advanced stage (III and IV) in comparison with early stage (I and II) *p* = 0.022. Sherief et al., Dinand et al., and Smith et al. reported similar results, *p* = 0.006, *p* = 0.0001, *p* < 0.001, respectively [17, 25, 26]. Presence of B symptoms was borderline significantly associated with lower 5-year survival rate (*p* = 0.05) while it was significant in other studies, *p* = 0.005 [25], *p* < 0.001 [27], respectively. We did not find any significant association between histopathological subtypes and 5-year survival rate (*p* = 0.395). But Sherief et al. and Smith et al. found significant correlation between NS subtype and lower survival rate (*p* = 0.001) [17] and (*p* = 0.02) [26], respectively.

A previous study performed on a HL cohort in Saudi Arabia found that the xeroderma pigmentosum complementation group G (XPG) repair gene had statistically significant association with cHL patient survival [27]. The limitation of our study was that we did not correlate our results with genetic studies.

The association of DADA2 (deficiency of adenosine deaminase 2) with lymphoproliferation is well known [28]. In our study, we report familial HL in two children with a novel deleterious mutation in ADA2 and associated with DADA2. The details of both cases were reported before [29].

In our cohort, one patient developed secondary leukemia (AML). This 14-year-old patient was diagnosed with intermediate risk HL (stage IIIA, non-bulky), and was in remission for 54 months. He presented with fever, fatigue, lymphadenopathy, and hepatosplenomegaly. The BM and immunophenotype was compatible with AML (FAB: M0). A lymph node biopsy showed a myeloid sarcoma. The details were reported before [30].

## Conclusion

In the last few decades, the outcome of HL especially in pediatric age was improved considerably in the developing countries. Our study provided a unique descriptive study of childhood HL with valuable insight into the long-term outcome and late toxicity in Saudi Arabia. The efficacy of our management strategies illustrated an excellent EFS and OS. So, our results are comparable to other studies in the Middle East and European countries with a little difference in the prominent histopathological subtypes which may be related to the difference in genetic background in the gulf population.

## Data Availability

The data that support the findings of this study are available from a password-protected database in Prince Sultan Military Medical City, Riyadh, Saudi Arabia. But restrictions apply to the availability of these data, which were used under license for the current study, and so are not publicly available. Data are however available from the authors upon reasonable request and with permission of Prince Sultan Military Medical City.

## Abbreviations

HL: Hodgkin’s lymphoma
EFS: Event-free-survival
IRB: Institute review board
WHO: World Health Organization
CT: Computed tomography
PET: Positron emission tomography
CBC: Complete blood count
ABVD: Doxorubicin (adriamycin), bleomycin, vinblastine, and dacarbazine
COG: Children’s Oncology Group
COPP: Cyclophosphamide, vincristine, procarbazine, prednisone
R-CHOP: Rituximab-cyclophosphamide, hydroxydaunomycin, vincristine, and prednisolone
IFRT: Involved filed radiotherapy
ICE: Ifosfamide/carboplatin/etoposide
ESHAP: Etoposide/solu-medrol/high dose ara-c/platinum “cisplatin”
CR: Complete response
SER: Slow early responders
VGPR: Very good partial response
RER: Rapid early responders
SPSS: Statistical package for social sciences
CR1: Complete remission after first line therapy
CR2: 2Nd complete remission
cHL: Classic Hodgkin’s lymphoma
NLPHL: Nodular lymphocyte predominant Hodgkin lymphoma
R/R: Relapsed/refractory
OS: Overall survival
NS: Nodular sclerosis
MC: Mixed cellularity
LR: Lymphocyte rich
LD: Lymphocyte depleted
EBV: Epstein-Barr virus
DADA2: Deficiency of adenosine deaminase 2

## References

[1] Thomas RK, Re D, Zander T, Wolf J, Diehl V. Epidemiology and etiology of Hodgkin’s lymphoma. Ann Oncol. 2002;13(4):147–52. 10.1093/annonc/mdf652. 12401681 10.1093/annonc/mdf652

[2] Elborai Y, Elgammal A, Salama A, Fawzy M, El-Desouky ED, Attia I, et al. Cyclooxygenase-2 expression as a prognostic factor in pediatric classical Hodgkin lymphoma. Clin Transl Oncol. 2020;22:1539–47. 10.1007/s12094-020-02297-8. 31970686 10.1007/s12094-020-02297-8

[3] Chang ET, Zheng T, Weir EG, Borowitz M, Mann RB, Spiegelman D, et al. Childhood social environment and Hodgkin’s lymphoma: new findings from a population-based case-control study. Cancer Epidemiol Biomarkers Prev. 2004;13(8):1361–70. 15298959

[4] Glaser SL, Clarke CA, Nugent RA, Stearns CB, Dorfman RF. Social class and risk of Hodgkin’s disease in young-adult women in 1988–94. Int J Cancer. 2002;98(1):110–7. 10.1002/ijc.10164. 11857394 10.1002/ijc.10164

[5] Dinand V, Arya LS. Epidemiology of childhood Hodgkin’s disease: is it different in developing countries? Indian Pediatr. 2006;43(2):141–7. 16528110

[6] Jemal A, Siegel R, Ward E, Hao Y, Xu J, Thun MJ. Cancer statistics. CA Cancer J Clin. 2009;59(4):225–49. 10.3322/caac.20006. 19474385 10.3322/caac.20006

[7] Alhashmi H, Kandil M, Alhejazi A, Motabi I, Sagheir A, Alzahrani M, et al. Hodgkin’s lymphoma: Saudi Lymphoma Group’s clinical practice guidelines for diagnosis, management and follow-up. Saudi J Med Med Sci. 2019;7(3):195–201. 10.4103/sjmms.sjmms_96_19. 31543744 10.4103/sjmms.sjmms_96_19 PMC6734729

[8] Belgaumi AF, Pathan GQ, Siddiqui K, Ali AA, Al-Fawaz I, Al-Sweedan S, Ayas M, Al-Kofide AA. Incidence, clinical distribution, and patient characteristics of childhood cancer in Saudi Arabia: a population-based analysis. Pediatr Blood Cancer. 2019;66(6): e27684. 10.1002/pbc.27684. 30803142 10.1002/pbc.27684

[9] Fitzmaurice C, Allen C, Barber RM, Barregard L, Bhutta ZA, Brenner H, et al. Global, regional, and national cancer incidence, mortality, years of life lost, years lived with disability, and disability-adjusted life-years for 32 cancer groups, 1990 to 2015: a systematic analysis for the Global Burden of Disease Study. JAMA Oncol. 2017;3(4):524–48. 10.1001/jamaoncol.2016.5688. 27918777 10.1001/jamaoncol.2016.5688 PMC6103527

[10] Maggioncalda A, Malik N, Shenoy P, Smith M, Sinha R, Flowers CR. Clinical, molecular, and environmental risk factors for Hodgkin lymphoma. Adv Hematol. 2011:736261. doi: 10.1155/2011/736261. 10.1155/2011/736261 PMC2994062 21127715

[11] Oguz A, Karadeniz C, Okur FV, Citak EC, Pinarli FG, Bora H, et al. Prognostic factors and treatment outcome in childhood Hodgkin disease. Pediatr Blood Cancer. 2005;45(5):670–5. 10.1002/pbc.20487. 16007600 10.1002/pbc.20487

[12] Campo E, Swerdlow SH, Harris NL, Pileri S, Stein H, Jaffe ES. The 2008 WHO classification of lymphoid neoplasms and beyond: evolving concepts and practical applications. Blood. 2011;117(19):5019–32. 10.1182/blood-2011-01-293050. 21300984 10.1182/blood-2011-01-293050 PMC3109529

[13] Lister TA, Crowther D, Sutcliffe SB, Glatstein E, Canellos GP, Rosenberg RC, et al. Report of a committee convened to discuss the evaluation and staging of patients with Hodgkin’s disease: cotswolds meeting. J Clin Oncol. 1989;7(11):1630–6. 10.1200/JCO.1989.7.11.1630. 2809679 10.1200/JCO.1989.7.11.1630

[14] Hasenclever D, Diehl V. A prognostic score for advanced Hodgkin’s disease: international prognostic factors project on advanced Hodgkin’s disease. N Engl J Med. 1998;339(21):1506–14. 10.1056/NEJM199811193392104. 9819449 10.1056/NEJM199811193392104

[15] Cheson BD, Horning SJ, Coiffier B, Shipp MA, Fisher RI, Lister JM, et al. Report of an international workshop to standardize response criteria for non-Hodgkin’s lymphomas NCI Sponsored International Working Group. J Clin Oncol. 1999;17(4):1244. 10.1200/JCO.1999.17.4.1244. 10561185 10.1200/JCO.1999.17.4.1244

[16] Aquino S, Clavio M, Rossi E, Vignolo L, Miglino M, Spriano M, et al. Therapy of Hodgkin’s lymphoma in clinical practice: a retrospective long-term follow-up analysis. Oncol Lett. 2011;2:289–95. 10.3892/ol.2011.255. 22866079 10.3892/ol.2011.255 PMC3410564

[17] Sherief LM, Elsafy UR, Abdelkhalek ER, Naglaa MK, Rabab E, Tamer HH, et al. Hodgkin lymphoma in childhood, clinicopathological features and therapy outcome at 2 centers from a developing country. Medicine. 2015;94(15):670–6. 10.1097/MD.0000000000000670. 10.1097/MD.0000000000000670 PMC4602501 25881843

[18] Stefan DC, Stones D. How much does it cost to treat children with Hodgkin lymphoma in Africa? Leuk Lymphoma. 2009;50(2):196–9. 10.1080/10428190802663205. 19197725 10.1080/10428190802663205

[19] Baez F, Ocampo E, Conter V, Flores A, Gutierrez T, Malta A, et al. Treatment of childhood Hodgkin’s disease with COPP or COPP-ABV (hybrid) without radiotherapy in Nicaragua. Ann Oncol. 1997;8(3):247–50. 10.1023/a:1008200210674. 9137793 10.1023/a:1008200210674

[20] Feldman AL, Pittaluga S, Jaffe ES. Classification and histopathology of the lymphomas. In: Canellos GP, Lister TA, Young B, editors. The Lymphomas. 2nd ed. Philadelphia USA: Saunders Elsevier; 2006. p. 2–25.

[21] Pourtsidis A, Doganis D, Baka M, Bouhoutsou D, Varvoutsi M, Synodinou M, et al. Differences between younger and older patients with childhood Hodgkin lymphoma. Pediatr Hematol Oncol. 2013;30(6):532–6. 10.3109/08880018.2013.823471. 23941743 10.3109/08880018.2013.823471

[22] Arya LS, Dinand V, Thavaraj V, Bakhshi S, Dawar R, Rath GK, et al. Hodgkin’s disease in Indian children: outcome with chemotherapy alone. Pediatr Blood Cancer. 2006;46(1):26–34. 10.1002/pbc.20157. 16161019 10.1002/pbc.20157

[23] Audouin J, Diebold J, Nathwani B, Ishak E, MacLennan K, Mueller-Hermelink H, et al. Epstein-Barr virus and Hodgkin’s lymphoma in Cairo. Egypt J Hematop. 2010;3(1):11–8. 10.1007/s12308-010-0059-3. 21625283 10.1007/s12308-010-0059-3 PMC2883908

[24] Kamer Mutafoğlu Uysal, Riza Çetingöz, Dilek Güneş, Ayşe Demiral, Erdener Özer, Handan Çakmakçi, et al. Clinical characteristics and therapy outcome of pediatric Hodgkin’s lymphoma - a single centre experience from the west part of Turkey. Turkish J Cancer 2007;37(3):98–108.

[25] Dinand V, Dawar R, Arya LS, Unni R, Mohanty B, Singh R, et al. Hodgkin’s lymphoma in Indian children: prevalence and significance of Epstein-Barr virus detection in Hodgkin’s and Reed-Sternberg cells. Eur J Cancer. 2007;43(1):161–8. 10.1016/j.ejca.2006.08.036. 17113770 10.1016/j.ejca.2006.08.036

[26] Smith RS, Chen Q, Hudson MM, Link MP, Kun L, Billett HW, et al. Prognostic factors for children with Hodgkin’s disease treated with combined-modality therapy. J Clin Oncol. 2003;21(10):2026–33. 10.1200/JCO.2003.07.124. 12743158 10.1200/JCO.2003.07.124

[27] Al Sayed Ahmed H, Raslan WF, Deifalla AHS, Fathallah MD. Overall survival of classical Hodgkins lymphoma in Saudi patients is affected by XPG repair gene polymorphism. Biomed Rep. 2019;10(1):10–16. doi:10.3892/br.2018.1165. 10.3892/br.2018.1165 PMC6299211 30588297

[28] Alsultan A, Basher E, Alqanatish J, Mohammed R, Alfadhel M. Deficiency of ADA2 mimicking autoimmune lymphoproliferative syndrome in the absence of livedo reticularis and vasculitis. Pediatr Blood Cancer. 2018;65(4). doi: 10.1002/pbc.26912. 10.1002/pbc.26912 29271561

[29] Alabbas F, Elyamany G, Alsharif O, Hershfield M, Meyts I. Childhood Hodgkin lymphoma: think DADA2. J Clin Immunol. 2019;39(1):26–9. 10.1007/s10875-019-0590-7. 30644014 10.1007/s10875-019-0590-7

[30] Alkhayat N, Elyamany G, Elborai Y, Sedick Q, Al-Shahrani M, Al-Sharif O, et al. Rare cytogenetic abnormalities and their clinical relevance in pediatric acute leukemia of Saudi Arabian population. Mol Cytogenet. 2019;12:42–52. 10.1186/s13039-019-0454-0. 31632455 10.1186/s13039-019-0454-0 PMC6788108

